# Circulating Conjugated and Unconjugated Vitamin D Metabolite Measurements by Liquid Chromatography Mass Spectrometry

**DOI:** 10.1210/clinem/dgab708

**Published:** 2021-09-27

**Authors:** Carl Jenkinson, Reena Desai, Malcolm D McLeod, Jonathan Wolf Mueller, Martin Hewison, David J Handelsman

**Affiliations:** 1 Andrology, ANZAC Research Institute, University of Sydney, Sydney NSW 2139, Australia; 2 Institute of Metabolism and Systems Research, University of Birmingham, Birmingham, UK; 3 Research School of Chemistry, Australian National University, Canberra, Australian Capital Territory 2601, Australia

**Keywords:** Vitamin D, Hydrolysis, Conjugate, LC-MS/MS

## Abstract

**Context:**

Vitamin D status is conventionally defined by measurement of unconjugated circulating 25-hydroxyvitamin D (25OHD), but it remains uncertain whether this isolated analysis gives sufficient weight to vitamin D’s diverse metabolic pathways and bioactivity. Emerging evidence has shown that phase II endocrine metabolites are important excretory or storage forms; however, the clinical significance of circulating phase II vitamin D metabolites remains uncertain.

**Objective:**

In this study we analyzed the contribution of sulfate and glucuronide vitamin D metabolites relative to unconjugated levels in human serum.

**Methods:**

An optimized enzyme hydrolysis method using recombinant arylsulfatase (*Pseudomonas aeruginosa*) and beta-glucuronidase (*Escherichia coli*) was combined with liquid chromatography mass spectrometry (LC-MS/MS) analysis to measure conjugated and unconjugated vitamin D metabolites 25OHD3, 25OHD2, 3-epi-25OHD3, and 24,25(OH)2D3. The method was applied to the analysis of 170 human serum samples from community-dwelling men aged over 70 years, categorized by vitamin D supplementation status, to evaluate the proportions of each conjugated and unconjugated fraction.

**Results:**

As a proportion of total circulating vitamin D metabolites, sulfate conjugates (ranging between 18% and 53%) were a higher proportion than glucuronide conjugates (ranging between 2.7% and 11%). The proportion of conjugated 25OHD3 (48 ± 9%) was higher than 25OHD2 conjugates (29.1 ± 10%) across all supplementation groups. Conjugated metabolites correlated with their unconjugated forms for all 4 vitamin D metabolites (r = 0.85 to 0.97).

**Conclusion:**

Sulfated conjugates form a high proportion of circulating vitamin D metabolites, whereas glucuronide conjugates constitute a smaller fraction. Our findings principally in older men highlight the differences in abundance between metabolites and suggest a combination of both conjugated and unconjugated measurements may provide a more accurate assessment of vitamin D status.

Phase I metabolism of vitamin D represented by various hydroxylated metabolites has been extensively characterized, arriving at a consensus for phase I pathways and circulating concentrations (1, 2). Conventionally, vitamin D status is evaluated by measuring circulating levels of 25-hydroxyvitamin D (25OHD), the sum of 25OHD2 and 25OHD3, which circulate at stable ng/mL concentrations due to their long half-life of several weeks (3, 4). Both 25OHD3 and 25OHD2 are metabolized to bioactive 1,25-dihydroxy metabolites that circulate at much lower (pg/mL) concentrations. Inactivating hydroxylation of 25OHD3 occurs at the C-24 position to form 24,25(OH)_2_D3. Another phase I metabolic conversion, the C-3 epimerization of 25OHD3 and 25OHD2, has been characterized in forming 3-epi-25OHD3 or D2 which circulates at about 5% to 10% of 25OHD levels (1, 5).

Phase II metabolism of vitamin D is less well characterized and comprises largely hepatic conjugation reactions adding a sulfate ester by sulfotransferase (SULT) enzymes or a glucuronide moiety by UDP-glucuronosyltransferase enzymes (6, 7). These conjugation reactions inactivate the vitamin D metabolite and render it more hydrophilic, facilitating renal excretion (8-10). Circulating levels of conjugated vitamin D metabolites have not been well described, although a sulfated form of 25OHD3 (25OHD3-S) in levels that exceed circulating 25OHD3 has been reported (11, 12). This pattern has been confirmed by more recent liquid chromatography tandem mass spectrometry (LC-MS/MS) methods that have also reported a 25OHD3 glucuronide form (25OHD3-G) in circulation at low ng/mL levels (13, 14).

Both sulfate and glucuronide conjugates can undergo deconjugation back to their unconjugated forms by sulfatase and beta-glucuronidase enzymes, respectively. Both deconjugation enzymes have biological roles in local regulation of steroid action within tissues (10, 15) and are also used in analytical measurements for steroid profiling in sports antidoping tests (16, 17). Although direct measurements of conjugates have become feasible in recent years, analytical procedures including an effective deconjugation step are preferred as this enables a single-step analytical procedure for combined measurements of both unconjugated and conjugated forms. However, the biological role and application of hydrolysis enzymes in the analysis of circulating vitamin D is not known and to our knowledge there are no reported methods for measuring circulating phase II vitamin D metabolites by enzyme hydrolysis. As a result, it is not known whether combined unconjugated and conjugated serum measurements could be a more reliable approach for assessing vitamin D status instead of current methods. To address this, we developed enzyme hydrolysis methods in this study to evaluate the proportions of both conjugated and unconjugated forms of circulating vitamin D metabolites. While circulating phase I metabolites generally increase following supplementation with vitamin D (18), it is currently unclear what impact supplementation has on conjugation and the concentrations of phase II metabolites. In this study we therefore employed a composite hydrolysis assay using arylsulfatase enzyme variants and beta-glucuronidase prior to LC-MS/MS analysis to provide a comprehensive simultaneous measurement of circulating levels of conjugated and unconjugated forms of vitamin D, notably 25OHD3, 25OHD2, 3-epi-25OHD3, and 24,25(OH)_2_D3, in human serum samples and according to vitamin D supplementation status.

## Materials and Methods

### Chemicals and Consumables

Reference vitamin D standards (Supelco brand) 25OHD3, 25OHD2, 3-epi-25OHD3, and 24,25(OH)_2_D3 as well as corresponding deuterated internal standards 25OHD3-deuterated(d)3, 25OHD2-d3, 3-epi-25OHD3-d3, 24,25(OH)_2_D3-d6, and 25OHD3-S-d6 were purchased from Sigma Aldrich. The conjugate metabolites 25OHD3-3-S and 25OHD3-3-G were purchased from Toronto Research Chemicals. The derivatization reagent 4-phenyl-1,2,4-triazole-3,5-dione (PTAD) was purchased from Sigma Aldrich. The derivatization reagent 4-[2-(3,4-dihydro-6,7-dimethoxy-4-methyl-3-oxo-2-quinoxalinyl)ethyl]-3H-1, 2,4-triazole-3,5(4H)-dione (DMEQ-TAD) was purchased from R-Biopharm. Oasis HLP solid phase extraction (SPE) cartridges and a Waters ACQUITY BEH phenyl column (1.7 μm 2.1 × 75 mm) were purchased from Waters Corporation. LC-MS grade water, isopropanol, acetonitrile, and formic acid were purchased from Chem Supply Ltd. LC-MS grade methanol was purchased from Merck. Methyl *tert*-butyl ether was purchased from RCI Labscan Limited. A SecurityGuard ULTRA cartridge and holder for ultra-high performance liquid chromatography phenyl 2.1 mm ID columns was purchased from Phenomenex. Vitamin D metabolites in frozen human serum (SRM 972a) were purchase from the National Institute of Standards and Technology (Gaithersburg, USA). Vitamin D–depleted mass spectrometry-certified serum was purchased from Golden West Biologicals (USA). Beta-glucuronidase from *Escherichia coli* and Tris Cl buffer were purchased from Sigma Aldrich. Three *Pseudomonas aeruginosa* arylsulfatase enzyme variants (WT-PaS, PVFV-PaS, and DRN-PaS) were expressed in *E. coli* and purified as previously described (17, 19). PVFV-PaS was optimized for the hydrolysis of testosterone sulfate and DRN-PaS was optimized for the hydrolysis of etiocholanolone sulfate.

### Sample Preparation

**Figure 1.**
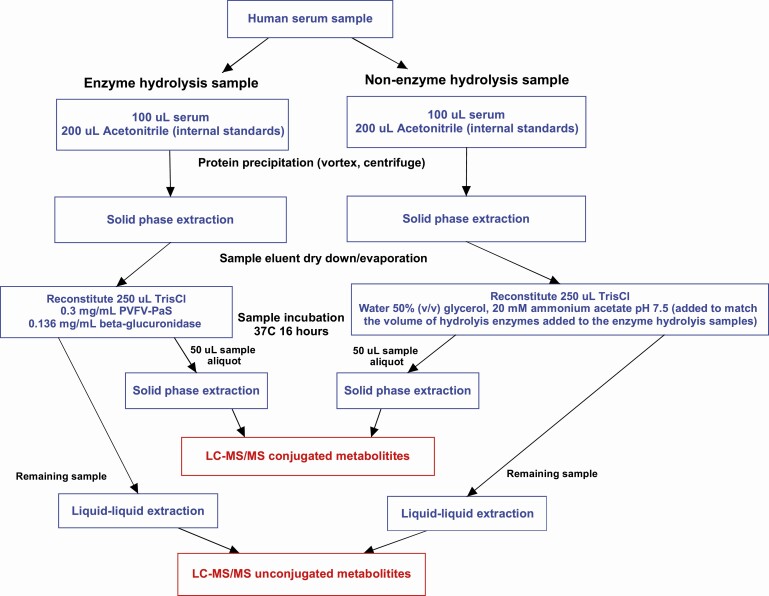
Sample preparation steps for each serum sample prior to LC-MS/MS analysis of vitamin D metabolites. The steps are outlined for samples with and without hydrolysis.

An overview of the sample preparation steps prior to LC-MS/MS analysis are shown in Fig. 1 and described in detail below.

### Prehydrolysis Sample Extraction

Initial extraction from serum of both conjugated and unconjugated vitamin D metabolites was by SPE as previously described (20) with modifications (Fig. 1). Prior to the SPE extraction, 100 µL serum samples underwent protein precipitation by adding 200 µL of acetonitrile solution containing the internal standards 25OHD3-d3 (20 ng/mL), 25OHD2-d3 (5 ng/mL), 3-epi-25OHD3-d3 (8 ng/mL), and 24,25(OH)_2_D3-d6 (8 ng/mL). Samples were vortexed for 1 minute, centrifuged at 1000*g* for 10 minutes then diluted with 550 µL of LC-MS grade water with the sample supernatant transferred for SPE. Oasis HLB SPE cartridges, primed by washing with 1 mL of LC-MS grade water, followed by 1 mL of LC-MS grade water/methanol (50/50), were loaded with sample supernatant followed by washing through with 1 mL of LC-MS grade water. Extracts were washed from the SPE cartridges by adding 1.5 mL of LC-MS grade ethyl acetate (to elute unconjugated metabolites) and then 1.2 mL of LC-MS grade methanol (to elute conjugate metabolites). The combined eluates were dried down under nitrogen gas at 50°C and reconstituted with 250 µL of 50mM Tris Cl buffer pH 7.4 and transferred to 1.5-mL microcentrifuge tubes for enzyme hydrolysis.

### Sample Hydrolysis

Enzymatic hydrolysis of vitamin D metabolites was performed by a previously described method optimized for the hydrolysis of steroid sulfate metabolites (17) as well as a previously described method for beta-glucuronidase hydrolysis (21). The optimization of the hydrolysis method for 0.3 mg/mL PVFV-PaS and 0.136 mg/mL beta-glucuronidase is detailed in the online repository (22). Both PVFV-PaS and beta-glucuronidase were added to samples in Tris Cl buffer (in capped 1.5-mL tubes) then inverted and incubated at 37°C for 16 hours. Controls without arylsulfatase or beta-glucuronidase enzymes but with added water (containing 50% (v/v) glycerol and 20 mM ammonium acetate pH 7.5) to equate volumes were treated in the same manner.

### Posthydrolysis Extraction

Hydrolyzed or control samples were further extracted for analysis of unconjugated and conjugated vitamin D metabolites by LC-MS/MS. A 50-µL aliquot was taken from each sample for SPE of conjugated metabolites, while the remaining sample volume was used for the liquid–liquid extraction (LLE) of unconjugated vitamin D metabolites performed as described (23). For the LLE of samples, an initial protein precipitation was performed by the addition of 450 µL of isopropanol/water (50/50) to samples and vortex mixing (15 minutes), followed by centrifugation (9000 rpm for 5 minutes). Sample supernatants were then transferred to glass tubes and 1 mL of LC-MS grade hexane was added to samples followed by 1 mL of LC-MS grade methyl *tert*-butyl ether. The separated organic layer was transferred to glass tubes and dried under nitrogen gas at 50°C and derivatization was performed by the addition of 0.125 mg/mL of the derivatization reagent 4-phenyl-1,2,4-triazole-3,5-dione dissolved in acetonitrile for 2 hours at room temperature in darkness. Derivatization was quenched by the addition of 20 µL of water and samples were dried under nitrogen gas at 50°C and reconstituted in 80 µL of methanol/water (50/50), then transferred to a 96-well microtiter plate for LC-MS/MS analysis.

The SPE extraction method for the analysis of 25OHD3-S and 25OHD-G was performed as previously described (14) with modifications. A 50 µL acetonitrile solution containing 25OHD3-S-d6 (20 ng/mL) was added to the 50 µL sample aliquots followed by vortex mixing for 30 seconds and centrifugation (10 000*g* for 10 minutes). Samples were then diluted with 600 µL of water prior to SPE extraction. SPE cartridges were primed by washing with 1 mL of LC-MS grade water followed by 1 mL of LC-MS grade water/methanol (50/50). Samples were then loaded onto SPE cartridges followed by washing with 1 mL of LC-MS grade water followed by sample elution with 1.2 mL of methanol. Samples were dried under nitrogen gas at 50°C and derivatization was performed by the addition of 30 µL of 0.1 mg/mL DMEQ-TAD dissolved in ethyl acetate, followed by the addition of a further 30 µL after 30 minutes. The reaction was terminated 60 minutes later by the addition of 40 µL of ethanol and samples were dried under nitrogen gas at 50°C and reconstituted in 80 µL of methanol/water (50/50). Samples were then transferred to 96-well microtiter plates for LC-MS/MS analysis.

### LC-MS/MS Conditions

LC-MS/MS analysis of the unconjugated analytes 25OHD3, 25OHD2, 3-epi-25OHD3, and 24,25(OH)_2_D3 was performed using a validated method (23). Calibration series were prepared in vitamin D–depleted serum across the following concentration ranges: 25OHD3 0.125 to 256 ng/mL, 25OHD2 0.063 to 128 ng/mL, 3-epi-25OHD3 0.125 to 32 ng/mL, 24,25(OH)_2_D3 0.125 to 32 ng/mL.

Direct analysis of the conjugated metabolites 25OHD3-S and 25OHD3-G was based on a previously described method (14) which was optimized with modifications described in the online repository (22). Analysis was performed on a SCIEX Exion LC system couple to a SCIEX 6500 QTRAP mass spectrometer, using electrospray ionization in negative mode. A Waters UPLC BEH Phenyl (2.1 × 75 mm, 1.7 µm) column was used for liquid chromatography separation. The column temperature was set to 40°C and the flow rate was 0.400 mL/minute with a mobile phase consisting of A, water 0.1% formic acid; B, methanol 0.1% formic acid with the following mobile phase gradient: 0 minutes, 50%-A:50%-B; 0.50 to 3 minutes, 1%-A:99%-B; 3.0 to 4.0 minutes, 0%-A:100%-B; 4.0 to 4.5 minutes, 0%-A:100%-B; 4.5 to 5.5 minutes, 50%-A:50%-B. The overall run time was 7 minutes. Calibration series were prepared in vitamin D–depleted serum across the following concentration ranges; 25OHD3-S 1-128 ng/mL, 25OHD2 0.125-32 ng/mL.

For both LC-MS/MS methods the generation of calibration curves for data acquisition and processing was performed using Analyst 1.6.3 software (AB SCIEX). Calibration curves were generated by plotting the peak area ratios of each analyte over internal standard against the respective analyte concentration (fit: linear, weight: 1/x).

### Method Validation

Quality control samples for the optimization and validation of methods were prepared by spiking known concentrations of unconjugated and conjugated vitamin D metabolites in vitamin D–depleted serum. The limit of detection (LOD) and lower limit of quantitation (LLOQ) were determined as per as per FDA and EMEA guidelines by the lowest concentration of each analyte that gave a signal-to-noise ratio of at least 3 (LOD) and 10 (LLOQ). The LLOQ was the lowest calibration standard used and had coefficient of variation (CV)% values <20% across 4 replicate QC measurements (24, 25). The accuracy and precision of the LC-MS/MS analysis of unconjugated and conjugated metabolites was performed by extracting QC samples across 3 concentration levels (low, medium, and high) for each analyte. The precision (CV%) value of each analyte was calculated from the measurements of 5 replicates for each QC level. The accuracy of each analyte was calculated from the mean of 5 replicates from each QC level against the nominal analyte concentration. We assessed the accuracy and precision initially after the prehydrolysis SPE step described above, and then after the completion of the entire sample preparation procedure: prehydrolysis SPE plus sample incubation for 16 hours at 37°C and LLE. To assess the accuracy of the method across sample batches, NIST 972a vitamin D metabolites in human serum samples were run and the measurements were compared with the NIST-certified reference levels.

The extraction recoveries of unconjugated and conjugated metabolites were performed and calculated as previously described by Matuszewski et al. (26). Extraction recovery was assessed by comparing the peak area of analytes and internal standards spiked in a solution of water/methanol (50/50) at the same concentrations as extracted QC samples. Five water/methanol (50/50) samples and 5 QC samples were measured across 3 concentration ranges (low, medium, and high). The resulting concentrations were then compared between the QC extracted samples and the analytes spiked in methanol/water (50/50). Two sets of these extraction recoveries were performed. Initially we assessed the extraction recovery after the first prehydrolysis SPE step described above. We then completed a second extraction recovery after the entire sample preparation procedure was completed that included the prehydrolysis SPE as well as a 16-hour 37°C sample incubation step and LLE or SPE.

Validation of the hydrolysis method was performed using QC samples containing known concentrations of 25OHD3-S and 25OHD3-G at 3 different concentration levels (low, medium, and high). Enzyme hydrolysis was performed with 6 replicate samples from each QC level by PVFV-PaS and beta-glucuronidase enzymes. In addition, the enzyme hydrolysis reaction was performed with WT-PaS and DRN-PaS using 3 replicate samples from each QC level. Following enzyme hydrolysis, the unconjugated 25OHD3 was measured to determine the abundance of 25OHD3-S or 25OHD3-G that had been deconjugated.

### Analysis of Serum

The validated method was used to measure vitamin D metabolites in serum samples obtained from 5 healthy human volunteers (aged 27-71, 3 female, 2 male). The analysis of unconjugated and conjugated vitamin D metabolites was performed for each of the 5 subjects after the following enzyme hydrolysis conditions: PVFV-PaS only, beta-glucuronidase only, PVFV-PaS and beta glucuronidase combined, no hydrolysis enzymes present. The vitamin D metabolite measurements were used to compare changes in deconjugation across each of the enzyme hydrolysis conditions and determine the proportion of conjugated and unconjugated metabolites in these samples.

Samples (n = 170) from the Concord Health and Ageing in Men Project (CHAMP), consisting of population-representative cohort of community-dwelling men aged 70 and over (27) were used to measure vitamin D metabolite levels in each sample with and without hydrolysis. Samples were grouped based on vitamin D supplementation status: nonsupplement (n = 89), D3 supplemented (n = 57), D2 supplemented (n = 24) (providing 25 µg/1000 IU of vitamin D per day). Supplementation status was determined by questionnaire as described previously (27-29) with supplements including ergocalciferol (D2), cholecalciferol (D3). Vitamin D2 supplementation was verified by determining 25OHD2 values that were above the 97.5% CI for 25OHD2 measurements in nonsupplemented samples. Each serum sample was extracted separately twice as outlined in Fig. 1, once without and then with hydrolysis enzymes PVFV-PaS and beta-glucuronidase. After SPE both samples were incubated for 16 hours at 37°C and following further extraction the unconjugated and conjugated metabolites were measured. The metabolite concentrations were than compared in samples with and without hydrolysis enzymes, and across vitamin D supplementation groups.

### Data Analysis

One-way analysis of variance was used for equal and unequal variances for multiple comparisons of metabolite concentrations between different supplementation groups. Regression was using Passing-Bablok or linear regression with residual standard error used to define scatter around the regression and Spearman’s Rho for correlations between variables. The percentage fraction of unconjugated and/or conjugated forms in samples was determined by dividing the unconjugated (determined without hydrolysis) or conjugated measurements (determined after hydrolysis) × 100 by the total (combined unconjugated and conjugate) metabolite measurements.

## Results

### Validation Results

The LOD (ng/mL) and LLOQ obtained for each analyte were 25OHD3 (0.0625) 0.125 ng/mL, 25OHD2 (0.125) 0.250 ng/mL, 3-epi-25OHD3 (0.125) 0.250 ng/mL, 24,25(OH)_2_D3 (0.0312) 0.0625 ng/mL, 25OHD3-S 90.300) 0.500 ng/mL, and 25OHD3-G (0.0625) 0.125 ng/mL. Accuracy and precision values for unconjugated and conjugated vitamin D metabolite measurements are displayed in the online repository (Table S1 (22)). After the prehydrolysis SPE the vitamin D metabolite accuracy measurements in QC samples were within the ±15% target range for all analyte concentration ranges and the CV% precision values within ±15%. The accuracy QC measurements performed after all sample preparation steps, prehydrolysis SPE plus 37°C sample incubation for 16 hours and LLE, were also within the ±15% target range and the precision CV% values were within ±15% (Table S1 (22)). The accuracy results for NIST972a sample measurements are displayed in the online repository (Table S2 (22)) that showed each analyte in these samples was measured within ±15% of the NIST certified reference concentrations. Extraction recovery ranged between 85% and 129% (Table S1 (22)).

Enzymatic hydrolysis results demonstrated complete deconjugation by 2 arylsulfatase variants WT-PaS and PVFV-PaS of 25OHD3-S as well as complete deconjugation by beta-glucuronidase of 25OHD3-G (Table 1). The arylsulfatase variant DRN-PaS displayed incomplete hydrolysis of 25OHD3-S in samples as the proportion of 25OHD3-S converted after hydrolysis ranged between 48.8% and 81.5%.

**Table 1.** Measurements of unconjugated 25OHD3 in QC samples after hydrolysis of 25OHD3-S by 3 arylsulfatase enzymes and 25OHD3-G by beta-glucuronidase

	25OHD3 (ng/mL)		
Hydrolysis enzyme	QCL (25OHD3-S 2.5 ng/mL)	QCM (25OHD3-S 10 ng/mL)	QCH (25OHD3-S 60 ng/mL)
PVFV (n = 6)	2.52 ± 0.09 (100.8%)	10.82 ± 0.30 (102.8%)	59.2 ± 1.09 (98.7%)
WT (n = 3)	2.56 ± 1.22 (102.4%)	10.65 ± 0.22 (106.5%)	60.1 ± 1.24 (100.2%)
DRN (n = 3)	1.22 ± 0.02 (48.8%)	7.74 ± 0.27 (77.4%)	48.9 ± 1.50 (81.5%)
	QCL (25OHD3-G 0.25 ng/mL)	QCM (25OHD3-G 1 ng/mL)	QCH (25OHD3-G 15 ng/mL)
Beta-glucuronidase (n = 6)	0.253 ± 0.018 (101.2%)	1.10 ± 0.12 (110.0%)	15.1 ± 0.36 (100.7%)

Data expressed as mean ± standard deviation (% conversion).

### Application of Beta-glucuronidase and PVFV-PaS in Human Serum Samples

LC-MS/MS analysis of 5 human serum samples revealed increased concentrations of the vitamin D metabolites 25OHD3, 25OHD2, 3-epi-25OHD3, and 24,25(OH)_2_D3 after hydrolysis by beta-glucuronidase and PVFV-PaS compared with the samples without either enzyme present (Table 2). The most abundant vitamin D metabolite form in samples was 25OHD3 while the other metabolites 25OHD2, 3-epi-25OHD3, and 24,25(OH)_2_D3 were all lower in concentration in all sample hydrolysis conditions. The proportion of unconjugated 25OHD3 in samples was 57 ± 7% whereas unconjugated 25OHD2 was 68 ± 9% and unconjugated 3-epi-25OHD3 and 24,25(OH)_2_D3 in samples were 71 ± 6.5% and 44 ± 6.7%, respectively.

**Table 2.** Serum concentrations of 25OHD3, 25OHD2, 3-epi-25OHD3, and 24,25(OH)_2_D3 in 5 human serum samples under different hydrolysis conditions

	25OHD3 (ng/mL)			25OHD2 (ng/mL)		
Serum	No enzyme	Beta-glucuronidase	PVFV-PaS	No enzyme	Beta-glucuronidase	PVFV-PaS
S1	31.1 (64.1%)	31.4 (0.6%)	48.2 (35.3%)	0.351 (72.2%)	0.389 (7.8%)	0.448 (20.0%)
S2	20.9 (53.7%)	22.2 (3.3%)	37.6 (42.9%)	0.460 (63.2%)	0.486 (3.6%)	0.702 (33.2%)
S3	12.7 (65.1%)	13.1 (2.1%)	19.1 (32.8%)	0.588 (76.2%)	0.594 (0.8%)	0.766 (23.1%)
S4	23.5 (48.0%)	26.2 (5.5%)	46.3 (46.5%)	0.310 (74.7%)	0.314 (1.0%)	0.411 (24.3%)
S5	34.1 (55.7%)	36.3 (3.6%)	59.0 (40.7%)	0.783 (54.4%)	0.853 (4.9%)	1.370 (40.8%)
**Mean**	**24.5 ± 8.5 (57.3 ± 7.3%)**	**25.8 ± 8.9 (3.0 ± 1.8%)**	**42.0 ± 14.9 (39.6 ± 5.6%)**	**0.498 ± 0.192 (68.1 ± 9.2%)**	**0.527 ± 0.210 (3.6 ± 2.9%)**	**0.739 ± 0.385 (28.3 ± 8.6%)**
	3-Epi-25OHD3 (ng/mL)			24,25(OH)_2_D3 (ng/mL)		
Serum	No enzyme	Beta-glucuronidase	PVFV-PaS	No enzyme	Beta-glucuronidase	PVFV-PaS
S1	1.74 (66.4%)	2.11 (14.4%)	2.25 (19.5%)	1.47 (46.1%)	1.51 (1.3%)	3.15 (57.2%)
S2	1.48 (68.8%)	1.74 (12.1%)	1.89 (19.1%)	1.76 (38.3%)	1.82 (1.3%)	4.54 (60.4%)
S3	0.62 (63.3%)	0.77 (15.3%)	0.83 (21.4%)	0.83 (55.3%)	0.89 (4.0%)	1.44 (40.7%)
S4	1.47 (77.8%)	1.57 (5.3%)	1.79 (16.9%)	2.07 (41.2%)	2.33 (5.2%)	4.76 (53.6%)
S5	2.11 (77.3%)	2.33 (8.1%)	2.51 (14.7%)	2.34 (41.0%)	2.43 (1.6%)	5.62 (57.4%)
Mean	1.48 ± 0.55 (70.7 ± 6.5%)	1.70 ± 0.60 (11.0 ± 4.2%)	1.85 ± 0.64 (18.3 ± 2.6%)	1.69 ± 0.58 (44.4 ± 6.7%)	1.80 ± 0.63 (2.7 ± 1.8%)	3.90 ± 1.64 (53.0 ± 7.5%)

Sample measurements were performed without hydrolysis (No enzyme) or with either PVFV-PaS or beta-glucuronidase. The percentage of unconjugated and conjugated forms in each sample is shown in brackets; unconjugated % (no enzyme), glucuronide % (beta-glucuronidase), and sulfate % (PVFV-PaS).

The sulfate form 25OHD3-S is present in high abundance (40 ± 5.6%) and the glucuronide fraction 25OHD3-G is in much lower abundance (3.0 ± 1.8%). Similarly, for 25OHD2 there was a greater proportion of the sulfate form (28 ± 8.6%) than the glucuronide form (3.6% ± 2.9%). The proportion of the sulfate form of 24,25(OH)_2_D3 (53 ± 7.5%) also exceeded the glucuronide conjugate (2.7 ± 1.8%). The sulfated form of 3-epi-25OHD3 was in relatively lower abundance (18.3 ± 2.6%) and the proportion of the glucuronide (11 ± 4.2%) was relatively higher than the other vitamin D metabolites.

**Figure 2.**
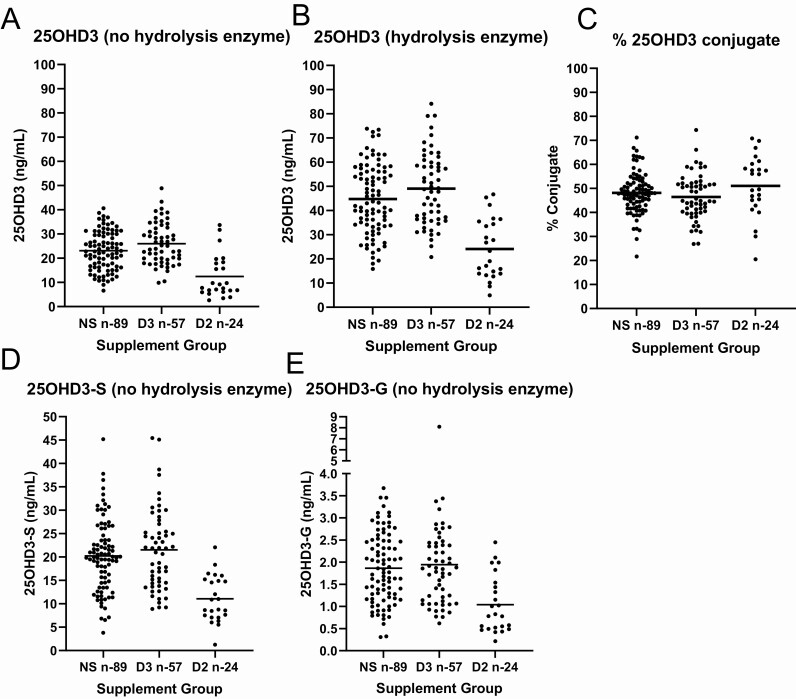
Serum concentrations of 25OHD3 metabolites in males aged +70 years categorized by vitamin D supplementation status; nonvitamin D supplemented (NS), vitamin D3 supplemented (D3), and vitamin D2 supplemented (D2). (A,D,E) represent the serum concentrations of 25OHD3, 25OHD3-S and 25OHD3-G in samples without hydrolysis. (B) represent serum concentrations of 25OHD3 after hydrolysis by beta-glucuronidase and PVFV-PaS. (C) represents the calculated percentage of conjugated forms of 25OHD3.

**Figure 3.**
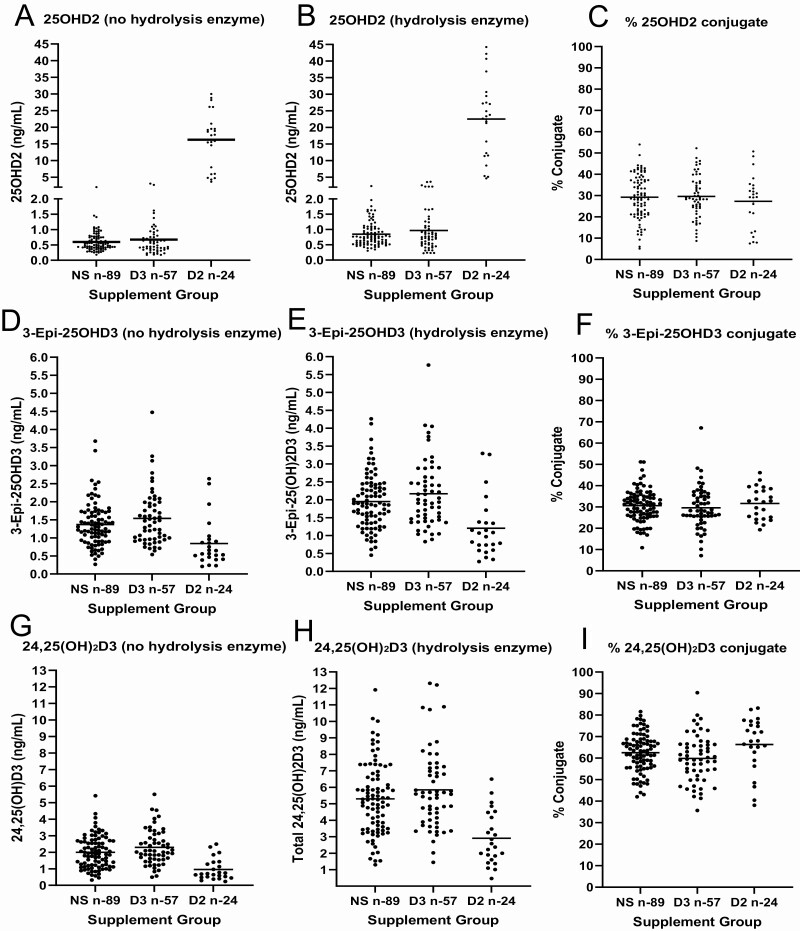
Serum concentrations of vitamin D metabolites in males aged +70 years categorized by vitamin D supplementation status; nonvitamin D supplemented (NS), vitamin D3 supplemented (D3), and vitamin D2 supplemented (D2). (A,D,G) represent the serum concentrations of 25OHD2, 3-epi-25OHD3 and 24,25(OH)_2_D3 in samples without hydrolysis. (B,E,H) represent serum concentrations of 25OHD2, 3-epi-25OHD3 and 24,25(OH)_2_D3 after hydrolysis by beta-glucuronidase and PVFV-PaS. (C,F,I) represent the calculated percentage of conjugated forms of 25OHD2, 3-epi-25OHD3 and 24,25(OH)_2_D3.

Vitamin D metabolites were measured in 170 samples by LC-MS/MS without and following hydrolysis with PVFV-PaS and beta-glucuronidase, with individual sample measurements of all 4 vitamin D metabolites shown in Fig. 2 (25OHD3) and Fig. 3 (3-epi-25OHD3, 24,25(OH)_2_D3, and 25OHD2). An overview of concentrations from the sample cohort when split into 3 groups according to vitamin D supplementation is shown in Table 3 (25OHD3) and Table 4 (3-epi-25OHD3, 24,25(OH)_2_D3 and 25OHD2).

**Table 3.** Serum concentrations of unconjugated, sulfate and glucuronide 25OHD3 and the percentage of 25OHD3 as conjugate forms in samples from men aged 70+ with and without vitamin D supplementation

	25OHD3 (ng/mL)			25OHD3-S (ng/mL)	25OHD3-G (ng/mL)
	No hydrolysis	Hydrolysis	% Conjugated	No hydrolysis	No hydrolysis
Nonsupplement N-89	23.1 ± 8.0 22.8 (16.6, 30.0)	44.8 ± 14.3 44.3 (34.1, 56.1)	48.2 ± 8.4%	20.2 ± 7.7 20.0 (14.5, 23.6)	1.86 ± 0.79 1.77 (1.21, 2.48)
D3 supplement N-57	26.0 ± 8.5 25.1 (19.7 32.3)	49.0 ± 15.0 48.8 (36.4, 59.4)	46.5 ± 9.4%	21.5 ± 8.7 21.9 (14.5, 25.4)	1.94 ± 1.10 1.86 (1.14, 2.38)
D2 supplement N-24	12.4 ± 8.8 8.5 (6.55, 18.0)	24.1 ± 12.4 21.2 (13.9, 34.7)	51.1 ± 12.7%	11.0 ± 4.97 9.85 (7.40,14.98)	1.04 ± 0.64 0.81 (0.51,1.47)

Data expressed as mean ± standard deviation, median (IQR 25%, 75%). Unconjugated fraction measured without hydrolysis (No hydrolysis) and combined unconjugated and conjugated fractions measured after hydrolysis by PVFV-PaS and beta-glucuronidase (Hydrolysis). 25OHD3-S and 25OHD3-G measurements performed in samples without hydrolysis. The percentage conjugated value is the proportion of 25OHD3 conjugate forms present in samples that was determined after hydrolysis.

**Table 4.** Serum concentrations of 3-epi-25OHD3, 24,25(OH)_2_D3, and 25OHD2 and the percentage of each analyte as conjugate forms in samples from men aged 70+ with and without vitamin D supplementation

	3-Epi-25OHD3 (ng/mL)			24,25(OH)_2_D3 (ng/mL)		
	No hydrolysis	Hydrolysis	% Conjugated	No hydrolysis	Hydrolysis	% Conjugated
Nonsupplement N-89	1.37 ± 0.61 1.30 (0.91, 1.71)	1.95 ± 0.76 1.89 (1.37, 2.39)	30.6 ± 7.5%	2.00 ± 0.97 1.91 (1.20, 2.68)	5.30 ± 2.16 5.25 (3.50, 6.60)	62.5 ± 9.0%
D3 supplement N-57	1.54 ± 0.75 1.43 (0.96, 1.96)	2.17 ± 0.95 1.98 (1.47, 2.62)	29.6 ± 9.7%	2.30 ± 1.06 2.07 (1.59, 2.86)	5.84 ± 2.42 5.58 (4.02, 7.17)	59.1 ± 11.0%
D2 supplement N-24	0.84 ± 0.66 0.60 (0.45, 0.91)	1.20 ± 0.83 1.02 (0.70, 1.36)	31.6 ± 7.5%	0.95 ± 0.65 0.69 (0.51, 1.31)	2.91 ± 1.61 2.47 (1.78, 4.24)	66.2 ± 12.6%
	25OHD2 (ng/mL)					
	No hydrolysis	Hydrolysis	% Conjugated			
Nonsupplement N-89	0.60 ± 0.30 0.52 (0.41, 0.75)	0.84 ± 0.40 0.75 (0.55, 1.03)	29.2 ± 10.2%			
D3 supplement N-57	0.67 ± 0.54 0.54 (0.35, 0.76)	1.0 ± 0.73 0.76 (0.53, 1.10)	29.6 ± 10.0%			
D2 supplement N-24	16.25 ± 8.35 17.68 (7.47, 19.90)	22.54 ± 11.59 23.00 (12.06, 27.98)	27.3 ± 12.5%			

Data expressed as mean ± standard deviation, median (IQR 25%, 75%). Unconjugated fraction measured without hydrolysis (No hydrolysis) and then as combined unconjugated and conjugated fractions measured after hydrolysis by PVFV-PaS and beta-glucuronidase (Hydrolysis). The percentage conjugated value is the proportion of conjugate forms present in samples that was determined after hydrolysis.

**Figure 4.**
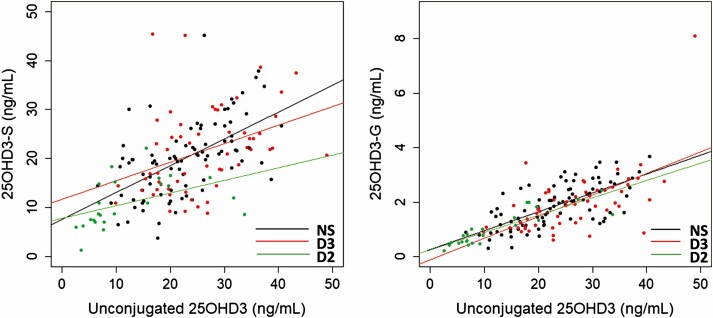
Correlations between circulating unconjugated 25OHD3 and 25OHD3-S, and unconjugated 25OHD3 and 25OHD3-G; NS, nonsupplemented (n = 89); D3, vitamin D3 supplemented (n = 57); D2, vitamin D2 supplemented (n = 24).

There was a significantly higher proportion of 25OHD3 conjugated in the D2-supplemented samples (51 ± 13%) than the D3 supplemented samples (47 ± 9.4%) (*P* < .05). Directly measured 25OHD3-S and 25OHD3-G in samples without hydrolysis correlated with unconjugated 25OHD3 in each supplementation group: 25OHD3-S: NS r = 0.61, D3 r = 0.43, D2 r = 0.67; all *P* < .001; 25OHD3-G: NS r = 0.70, D3 r = 0.59, D2 r = 0.85; all *P* < .001 (Fig. 4).

The 24,25(OH)_2_D3 metabolite had the highest proportion of conjugate forms compared with the other vitamin D metabolites. In D2-supplemented samples conjugated 24,25(OH)_2_D3 (66 ± 13%) was significantly higher than in the D3 supplement group (59 ± 11%) (*P* < .05). The proportion of conjugated 24,25(OH)_2_D3 (63 ± 9.0%) in the nonsupplement group did not differ between D2- and D3-supplemented groups. By contrast, the proportions of conjugated 3-epi-25OHD3 and 25OHD2 were lower than their unconjugated forms and consistent across supplementation groups.

Concentrations of 25OHD3, 3-epi-25OHD3, and 24,25(OH)_2_D3 vitamin D metabolites were higher in the D3 supplementation group than the nonsupplement (*P* < .05) and significantly lower in the D2 supplemented group than the nonsupplement (*P* < .05), and this was observed in samples without and with hydrolysis (Tables 3 and 4).

The concentrations of all 4 conjugated vitamin D metabolites correlated with the respective samples unconjugated measurements across all supplementation groups (online repository (22) Table S3, Figure S1). The vitamin D3 metabolites 3-epi-25OHD3 and 24,25(OH)_2_D3 correlated with 25OHD3 in samples; however, the proportion of these metabolites in samples changed after hydrolysis (online repository (22) Table S4, Figure S2). The proportion of 24,25(OH)_2_D3 to 25OHD3 in samples was increased after hydrolysis, whereas the proportion 3-epi-25OHD3 to 25OHD3 was decreased.

## Discussion

This study describes an optimized and validated enzyme hydrolysis assay using recombinant arylsulfatase and beta-glucuronidase combined with targeted multimetabolite LC-MS/MS analysis for a comprehensive comparison of circulating conjugated and unconjugated vitamin D metabolites. Initially we investigated whether 3 recombinant arylsulfatase enzyme variants had hydrolysis activity toward sulfated vitamin D metabolites. Two variants (WT-PaS and PVFV-PaS) showed complete desulfation whereas a third (DRN-PaS) was only partially effective. Furthermore, hydrolysis using beta-glucuronidase also showed complete hydrolysis of vitamin D glucuronide conjugates. Application of PVFV-PaS hydrolysis revealed that human serum samples have sulfated forms of vitamin D present at high proportions in circulation relative to the unconjugated forms. By contrast, the glucuronide conjugates contribute a much smaller proportion to the total measured circulating vitamin D metabolites. When both beta-glucuronidase and PVFV-PaS are used together with human serum, the proportions of circulating conjugated vitamin D metabolites 25OHD3, 25OHD2, 3-epi-25OHD3, and 24,25(OH)_2_D3 varies from 29% to 62%. Hence, circulating conjugate forms of vitamin D often match or exceed the more commonly measured unconjugated forms. The proportions of 25OHD3 and 24,25(OH)_2_D3 as conjugated forms also differ according to vitamin D supplementation status. These findings have clinical relevance for vitamin D measurements as the sulfate form is overlooked by conventional analytical estimation of vitamin D status, a limitation further aggravated by the impact of vitamin D supplementation. The combination of enzymatic hydrolysis with LC-MS/MS methods established in this study therefore provides important tools for investigating vitamin D in health and disease states by quantifying simultaneously both conjugated and unconjugated vitamin D metabolites.

Previous reports have shown that the vitamin D3 metabolites 3-epi-25OHD3 and 24,25(OH)_2_D3 correlate with increased amounts of circulating 25OHD3 (23). We confirmed these findings, although the proportion of conjugated 3-epi-25OHD3 and 24,25(OH)_2_D3 to 25OHD3 differed after hydrolysis because of the differences in the proportion of their conjugate forms. Hence measurement of unconjugated metabolites may not reflect the overall differences between the distribution of these conjugated and unconjugated D3 metabolites in circulation. These differences in conjugation status raise further doubt on measurements of unconjugated 25-hydroxyitamin D levels as a sole measure of vitamin D status. In addition to sulfation and glucuronidation, there may be additional esterified vitamin D conjugates (30) that could also contribute to net circulating vitamin D measurements; however, no other additional vitamin D conjugates are known to circulate at significant levels (31). Future studies investigating this by alkaline or esterase hydrolysis would be of interest to appraise any wider spectrum of conjugated metabolites in the assessment of vitamin D status.

Previous studies have reported that 25OHD3-S circulates at similar levels or exceeding the circulating levels of unconjugated 25OHD3 (11, 12, 32). It is also reported that 25OHD3-G is present in circulation at lower ng/mL concentrations (0.5-3 ng/mL) (13, 14). Our study provides a new perspective by measuring simultaneously the concentrations of both unconjugated and conjugated forms of 25OHD3. Our findings indicate that about 50% of 25OHD3 circulates as the sulfate conjugated form, a finding broadly in agreement with a previous study (14). The biological significance for such high circulating levels of conjugated 25OHD3-S is not clear. However, there is minimal renal excretion of the sulfated form as previous studies failed to identify significant amounts of 25OHD3-S in urine (33). This suggests that the sulfated conjugate is unlikely to serve solely as an excretory form of inactivated vitamin D. Alternatively, as 25OHD3-S binds with high affinity to the circulating vitamin D binding protein (DBP) (33), it may form a circulating reservoir available for future deconjugation to 25OHD3 and available to cells for hydroxylation to biologically active 1α,25(OH)_2_D3 as a paracrine mechanism modulating local vitamin D action in various tissues (33-35). A similar mechanism is reported for steroid sulfate metabolites such as DHEAS and estrone sulfate, which are major circulating forms and are deconjugated by steroid sulfatase for their activation within target tissues (10, 15, 36, 37). This study confirms that 25OHD3-S and other vitamin D sulfate metabolites are substrates for arylsulfatase. Future studies demonstrating 25OHD3-S substrate specificity for human steroid sulfatase would support the hypothesis that 25OHD-S could be deconjugated at local tissue sites due to the ubiquitous presence of steroid sulfatase in human tissues (15, 36).

The biological role of vitamin D glucuronide conjugates is not well understood although glucuronylation is generally considered to be a physiologically irreversible inactivation step, occurring predominantly in the liver to increase hydrophilicity and facilitate renal excretion (7, 38). However, there is also some limited presence of beta-glucuronidase within gut bacteria (10) that could have a role in the hydrolysis of glucuronide conjugates excreted in bile for reabsorption via enterohepatic circulation (39, 40). The present study indicates that circulating 25OHD3, 25OHD2, and 24,25(OH)_2_D3 glucuronide conjugates are present but at much smaller fractions than the sulfated forms. While our study has confirmed *E. coli* beta-glucuronidase hydrolysis activity for vitamin D, the process of glucuronylation is more likely to be an irreversible step for analyte excretion because beta-glucuronidase activity within most human tissues do not reach significant levels.

Compared with the other vitamin D metabolites, the proportion of 3-epi-25OHD3 circulating as conjugated metabolites was much lower in our samples. This is in broad agreement with a previous study by Yoshimura et al. that reported 3-epi-25OHD3-S in cord blood at lower levels than 3-epi-25OHD3 and also determined that in vitro SULT2A1 sulfation activity for 3-epi-25OHD3 was approximately one-tenth of that for 25OHD3 (20). A possible explanation for reduced SULT2A1 activity for 3-epi-25OHD3 is that sulfation of 25OHD3 predominantly occurs at the C-3 position (33) and the stereochemistry is altered to the beta configuration following C-3 epimerization by 3-epimerase (41, 42), altering enzyme substrate interactions. As 3-epi-25OHD3 and its dihydroxy metabolites are less biologically active than 25OHD3 (43), it remains unclear whether 3-epi-25OHD3-S could be utilized as a storage form or if conjugation of this metabolite is primarily to facilitate excretion as proposed for the other vitamin D sulfate moieties. While the arylsulfatase used in this study appears to show similar levels of activity toward sulfated 3-epi-25OHD3-S and 25OHD3-S, it remains to be determined whether there is a similar level of activity for human steroid sulfatase toward the hydrolysis of these analytes relevant to the proposed the biological significance of the sulfate forms.

Our measurements indicate that 25OHD2 conjugates circulate at lower levels than unconjugated 25OHD2 and that D2 supplementation does not appear to alter the proportion of conjugated metabolites. The proportion of 25OHD2 in conjugated form was also lower than in 25OHD3 conjugates for unknown reasons. Both 25OHD3 and 25OHD2 have 3-hydroxyl groups in the alpha configuration (44) and any structural differences occur away from the C-3 sulfation site (33). However, the reduced circulating conjugate forms of 25OHD2 could be explained by the fact that 25OHD2 has a reduced circulating half-life and lower binding affinity for DBP compared with 25OHD3 (44-46). It is also possible that 25OHD2-S could bind with less affinity to DBP than 25OHD3-S as it has previously been observed that 25OHD3-S has similar DBP binding affinity as 25OHD3 (33). Reduced 25OHD2-S binding affinity to DBP could lead to reduced reabsorption and therefore lower circulating levels compared with 25OHD3-S.

We observed that the conjugated forms of 24,25(OH)_2_D3 are in high abundance in circulation almost exclusively as the sulfate conjugate with minimal glucuronide fraction. The hydroxylation of 25OHD3 to 24,25(OH)_2_D3 by CYP24A1 is considered to be an inactivation step in the vitamin D3 metabolic pathway (1, 2) with circulating unconjugated 24,25(OH)_2_D3 correlating strongly with 25OHD3 measurements (23). Further metabolism of 24,25(OH)_2_D3 can occur to ultimately form calcitroic acid, a urinary and biliary excretory vitamin D product (1, 47). Prior to this 24,25(OH)_2_D3 is 1-hydroxylated to the more biologically active 1,24,25-trihydroxyvitamin D3 which circulates at low (<25 pg/mL) concentrations (48). The higher abundance of sulfated 24,25(OH)_2_D3 may be explained by the additional hydroxyl group at the C-24 position that could provide an additional site for sulfation. While it has previously been reported that the C-3 position is the main sulfation site for 25OHD3 (33), the sulfation positions of 24,25(OH)_2_D3 have not previously been determined; however, the target sites for SULT enzyme activity may be increased if sulfation occurs at both the C-3 and C-24 positions. Excreted 24,25(OH)_2_D3 has been detected in urine following hydrolysis by beta-glucuronidase (49), so the low circulating amounts of the glucuronide fraction of 24,25(OH)_2_D3 suggest that it is rapidly excreted into urine following glucuronylation. The binding affinity of 24,25(OH)_2_D3 to DBP is higher than that of 25OHD3 (46, 50) but the irreversible inactivation by 24 hydroxylation suggests it is unlikely to represent any storage form of vitamin D but rather an excretory product. The high proportion of conjugated 24,25(OH)_2_D3 conjugates in circulation could be explained by its increased binding to DBP leading to increased kidney reabsorption. While it is possible that 25OHD3-S could be utilized as a storage form for deconjugation at target tissue sites, the role of 24,25(OH)_2_D3-S in the circulation is less clear and it is generally considered an irreversibly inactive excretory form. Nevertheless, some studies have also described some isolated biological activities for 24,25(OH)_2_D3 including binding to FAM57B2 stimulating lactosylceramide synthesis which in turn promoted fracture healing in mice (51), along with stimulating growth plate development (52). Although the precise role of circulating 24,25(OH)_2_D3-S remains unclear, a possible mechanism could involve deconjugation by steroid sulfatase at target sites to undertake these biological actions. A further explanation for the high abundant 24,25(OH)_2_D3-S could be as a reservoir for deconjugation and further hydroxylation to more biologically active trihydroxyvitamin D3 metabolites; however, this remains to be determined.

In summary, we have demonstrated that the measurements of circulating conjugated fractions of 4 vitamin D metabolites in human serum display a high proportion of circulating conjugated forms of these metabolites, notably the sulfate fraction. These would be overlooked by conventional analytical methods that only measure unconjugated forms. Our findings in this study were in a population-representative cohort of community-dwelling men aged over 70. However, we also observed similarly high levels of the same conjugated vitamin D metabolites in a small cohort of younger male and female samples (Table 2), and further studies are required to determine how well these findings can be extrapolated to different sex and age ranges. The optimized hydrolysis method established in this study will be an important tool in these future studies to understand the precise mechanism of these conjugate metabolites by investigating changes in vitamin D conjugation in health and disease conditions and populations. This could then address the current hypothesis outlined for circulating phase II metabolites (18, 19), such as whether vitamin D conjugation, notably sulfation, represents a process for inactivation and excretion of more hydrophilic inactive metabolites, and/or provides a reservoir storage mechanism for subsequent deconjugation to bioactive forms. Despite previous studies reporting high circulating levels of 25OHD3-S and its potential role in human health, almost all vitamin D analytical measurements intended to evaluate vitamin D status continue to focus on measuring solely unconjugated 25OHD3 and 25OHD2. However, our findings highlight the potential importance of combining these measurements with the measurement of the conjugated forms, especially as the sulfate fraction may constitute a circulating vitamin D binding protein–associated reservoir for bioactive vitamin D. Hence fully assessing vitamin D status may be better reflected by considering the variations in vitamin D conjugation activities that may differ between various metabolites as well as different individuals and disease states.

## Data Availability

Some or all datasets generated during and/or analyzed during the current study are not publicly available but are available from the corresponding author on reasonable request.

## Abbreviations

25OHD: 25-hydroxyvitamin D
CV: coefficient of variation
DBP: vitamin D binding protein
LC-MS/MS: liquid chromatography tandem mass spectrometry
LLE: liquid–liquid extraction
LLOQ: lower limit of quantitation
LOD: limit of detection
SPE: solid phase extraction
SULT: sulfotransferase
